# Impacts of Individual Daily Greenspace Exposure on Health Based on Individual Activity Space and Structural Equation Modeling

**DOI:** 10.3390/ijerph15102323

**Published:** 2018-10-22

**Authors:** Lin Zhang, Suhong Zhou, Mei-Po Kwan, Fei Chen, Rongping Lin

**Affiliations:** 1 School of Geography and Planning, Sun Yat-sen University, Guangzhou 510275, China; zhanglin8@mail2.sysu.edu.cn (L.Z.); FeiFei17213454@163.com (F.C.); linrp3@mail2.sysu.edu.cn (R.L.); 2 Guangdong Provincial Engineering Research Center for Public Security and Disaster, Guangzhou 510275, China; 3 Department of Geography and Geographic Information Science, Natural History Building, 1301 W Green Street, University of Illinois at Urbana-Champaign, Urbana, IL 61801, USA; mpk654@gmail.com; 4 Department of Human Geography and Spatial Planning, Utrecht University, 3584 CB Utrecht, The Netherlands

**Keywords:** greenspace exposure, health, human mobility, physical activity, structural equation modeling, Guangzhou

## Abstract

Previous studies on the effects of greenspace exposure on health are largely based on static contextual units, such as residential neighborhoods, and other administrative units. They tend to ignore the spatiotemporal dynamics of individual daily greenspace exposure and the mediating effects of specific activity type (such as physical activity). Therefore, this study examines individual daily greenspace exposure while taking into account people’s daily mobility and the mediating role of physical activity between greenspace exposure and health. Specifically, using survey data collected in Guangzhou, China, and high-resolution remote sensing images, individual activity space for a weekday is delineated and used to measure participants’ daily greenspace exposure. Structural equation modeling is then applied to analyze the direct effects of individual daily greenspace exposure on health and its indirect effects through the mediating variable of physical activity. The results show that daily greenspace exposure directly influences individual health and also indirectly affects participants’ health status through physical activity. With respect to the total effects, daily greenspace exposure helps improve participants’ mental health and contributes to promoting their social health. It also helps improve participants’ physical health, although to a lesser extent. In general, the higher the daily greenspace exposure, the higher the physical activity level and the better the overall health (including physical, mental, and social health).

## 1. Introduction

Economic growth and urbanization can bring better living conditions, various opportunities (e.g., rapid development of the tertiary industry, employment opportunities, education and health care opportunities), and challenges (e.g., resource destruction, environmental pollution and frequent disasters). In the process, however, environmental problems are becoming increasingly serious, including a dramatic decrease in greenspace. Many urban dwellers today do not have easy access to and contact with various forms of greenspace (e.g., parks, green corridors, and functional green structures), including natural and artificial greenspace, which has negative impacts on human health and sustainable development in urban areas [1]. A report by the World Health Organization (WHO) showed that nearly 25% of the world’s diseases were caused by environmental factors. With the faster-than-ever pace of modern life, an increasing number of urban residents experience unfavorable environmental exposures that adversely affect their mental health and often lead to negative emotions [2,3]. In addition, the modern living environment often leads to the separation between individuals and families, as well as the reduction in social cohesion and interaction. Therefore, countries around the world have formulated the National Environment and Health Action Plan (NEHAP) [4,5], which emphasizes environmental benefits that help counteract these urban threats and improve people’s health outcomes [6,7]. Coincidentally, China has put forward the “Healthy China” initiative and highlighted the role of the environment in promoting national health and quality of life.

Urban greenspace has been associated with a wide range of health benefits. Decades of research has examined the direct effects of greenspace on people’s physical and mental health based on fixed contextual or areal units (e.g., census tracts, postcode areas and street network buffers). Some researchers suggested that residential green environment can help to regulate microclimate [8], purify the air [9], reduce noise pollution [10], and promote the quality of the residential environment [11,12]. All of these ecological benefits contributed to reducing the risk of obesity [13] and high blood pressure and diabetes [14], thereby improving physical health in general [15,16]. Meanwhile, a rapidly expanding literature showed that exposures to greenspace help to strengthen individual attention [17], enhance intelligence and inspiration [18,19], and promote self-awareness and ability to reinvent oneself [20,21]. Availability of ample greenspace has been found to have restorative [18] and stress-relieving qualities [22,23], and is recommended as an effective way to decrease violence and crime [24]. Wood et al. [25] and Akpinar et al. [26] indicated that better psychosocial status was not only associated with the quantity and accessibility of greenspace, but also with the functions and types of the greenspace people are exposed to. Berg et al. [27] found that the time spent on visits to greenspace should be considered individually, since it is a mediator in the relationship between greenspace and mental health. 

Previous research mainly focuses on the direct effects of greenspace on physical and mental health. Recently, the literature on greenspace and individual health has expanded to consider its effects on people’s physical activity. Although urban inhabitants typically benefit from superior access to medical technology, health care, and other services, these benefits are offset by their sedentary lifestyle and lack of physical activity [28,29]. Inadequate physical activity has been identified as a major risk factor of human health. Urban greenspace is now recognized as a suitable setting for physical activity and for its potential for promoting health outcomes. Some studies sought to examine the association between objectively measured greenspace, physical activity, and physical health. The results suggested that the provision of abundant urban greenspace may reduce the risk of obesity and promote physical health by increasing people’s physical activity level [30,31]. A series of studies on the relationship between greenspace and individual mental health outcomes showed that physical activity is likely to be a mediating factor in this relationship: Namely, residents with higher levels of access to greenspace, and thus more opportunities for physical activity, reported better stress-relieving effects, mental health, and well-being [30,32,33,34]. 

However, previous studies have rarely paid attention to the influence of daily greenspace exposure on social health and social interaction. Generally, the above studies analyzed the effects of greenspace on one or two dimension(s) of health (e.g., physical health, mental health, or social health) through the mediator of physical activity, but the different effects of greenspace on different dimensions of health were ignored. Thus, this paper focuses on the direct and indirect effects of objectively measured greenspace on three dimensions of health (physical health, mental health, and social health), and compares the different effects of greenspace on these health dimensions. Considering the definition of “greenspace” in previous studies that only includes “vegetation coverage” as too narrow, this paper extends the concept of “greenspace” to the broader notion of “greenspace exposure” by adding an activity dimension according to the concept of “environmental exposure science.” Greenspace exposure in this study encompasses the quantity, quality, and accessibility of greenspace for a person to meet the needs for a better urban environment and recreational activities in the actual geographic areas of his or her daily life. Among the indicators of greenspace selected in this paper, green vegetation contributes to improving the natural environment. Physical activity sites can provide more structured environments for social interactions and physical activity [35]. In addition, accessibility to greenspace also has an impact on physical activity and social interactions.

In addition, some qualitative and quantitative studies examined the relationship between the environment and health using fixed geographic or contextual units based on buffer areas around individuals’ residences [36,37,38] or administrative units, such as census tracts, postcode areas and street network buffers [39,40,41]. These studies presupposed that the most relevant areas affecting health were residential neighborhoods or residence-based buffer zones delimited in a variety of ways. This presupposition entails the view that people who live in the same contextual unit experience the same environmental impacts, regardless of where they actually work or undertake their daily activities. However, it is inappropriate to use static geographic units like census tracts to represent people’s true activity space that exerts contextual influence on their health, since there are considerable differences in people’s daily spatiotemporal behaviors, which may lead to their exposures to different areas beyond their residential neighborhoods [42,43,44]. Static geographic units cannot accurately represent people’s activity space, since they ignore human mobility and daily spatiotemporal behaviors [45,46,47]. Thus, human mobility cannot be neglected and it is essential to look beyond residential neighborhoods to take into account people’s environmental exposure in their daily activity space. Recently, Kwan [45,46,48] called our attention to the crucial role of human mobility and daily activity space in accurately assessing people’s environmental exposure through the notion of the uncertain geographic context problem (UGCoP). Several studies have provided important evidence on how the UGCoP may affect research findings in environmental health studies and the need to use geographic units or methods that capture people’s spatiotemporal activities [49,50,51,52]. For example, Zhao et al. [47] suggested that researchers should try to estimate the influence of various environmental exposure on individual health more accurately using contextual units that can capture people’s daily activities and travel. Therefore, this study seeks to identify and delineate residents’ activity space during the 24 h of a weekday to capture the real contextual areas that people are exposed to and interact with, in order to advance the analysis of how true environmental exposure level in people’s daily activity space affect their health. The reason for choosing only weekdays to study is that participants in this research are between 19 and 59 years of age and are employed (e.g., employees, employers) (note that students are excluded from the study), which may lead to routine and similar activities during weekdays. However, there may be considerable variations and irregularity in their activities in weekend days. Therefore, we selected to focus on participants’ daily activities in a weekday as their usual behaviors in this research, since they have more regular and frequent activities in weekdays, which took up most of their individual life when compared with weekends. In addition, given that where and when people spend their time differ from individual to individual, the spatiotemporal features of daily activities and their cumulative effects are also considered in this article. These features include activity types, activity locations and actual time spent in different areas.

Based on such considerations, this study takes advantage of the methods of human mobility research and constructs a conceptual framework for modeling the health benefit of individual daily greenspace exposure. A structural equation model is used to analyze the causal mechanisms among daily greenspace exposure, physical activity, and individual health based on questionnaire survey data and objectively measured data (e.g., deriving vegetation coverage using remote sensing images). Meanwhile, individual activity space is considered when analyzing how greenspace exposure influence people’s health behaviors and outcomes. Specifically, the article seeks to address the following questions: Is there any relationship among daily greenspace exposure, physical activity and individual health from the perspective of human mobility and the uncertain geographic context problem? Does individual daily greenspace exposure directly affect health, and does it also indirectly affect health through the mediating role of physical activity? How do specific elements of daily greenspace exposure and physical activity influence the different dimensions of individual health? This paper seeks to enrich and deepen our knowledge of health geography and spatiotemporal behaviors. Moreover, it has theoretical and practical value for urban planning, informing greenspace construction programs and strategies for achieving global environmental health. 

## 2. Study Design

### 2.1. Conceptual Framework and Hypotheses

**Figure 1:**
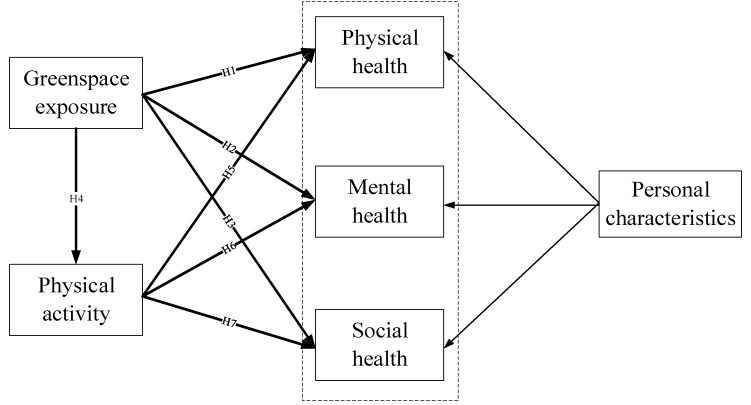
Conceptual framework.

Recent studies have examined the relationship among greenspace, physical activity and health, indicating that physical activity may play a partly intermediate role in this relationship [33,53,54]. Thus, the conceptual framework for this study focuses on the impacts of daily greenspace exposure and physical activity on individual health and is presented in Figure 1. As shown in the figure, the framework illustrates the interactions among daily greenspace exposure, physical activity, and individual health (physical health, mental health, and social health). On the one hand, daily greenspace exposure has a direct and important impact on health. On the other hand, it contributes to improving people’s overall health status indirectly through promoting physical activity, which plays a mediating role in the relationship between daily greenspace exposure and health outcomes. Thus, the following hypotheses are proposed based on this conceptual framework (Table 1). The paper will employ structural equation modeling to test these hypotheses.

### 2.2. Study Area

**Figure 2:**
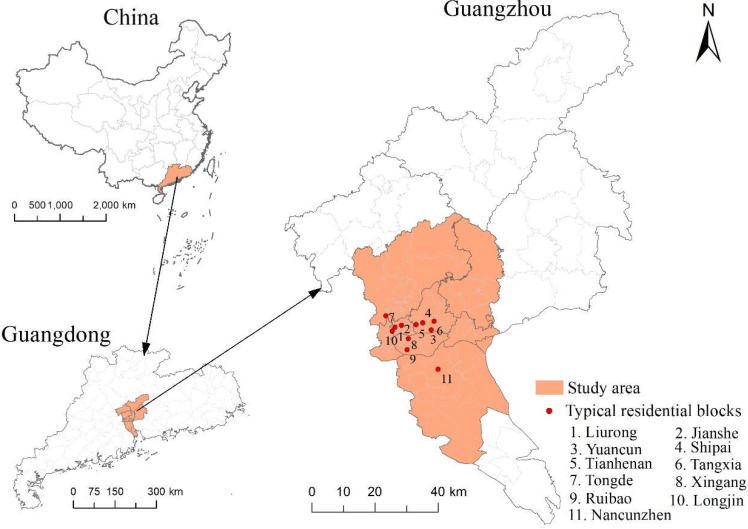
Study area.

The study area for this research is Guangzhou, China. As the capital of Guangdong Province, Guangzhou is one of the four megacities in China and has a total area of 7434.4 km^2^. The urbanization rate of Guangzhou reached 86.14% with a permanent population of about 14.5 million and a gross domestic product (GDP) of 2150.315 billion RMB in 2017 [55]. Increasing urbanization has resulted in a great proportion of the megacity’s population being exposed to environmental threats. This study thus selected Guangzhou as a representative megacity of China and investigated 11 typical residential blocks in it (Liurong, Jianshe, Yuancun, Shipai, Tianhenan, Tangxia, Tongde, Xingang, Ruibao, Longjin and Nancunzhen). Each of these residential blocks is nearly 1 km^2^ in area, and they include historical blocks, *danwei* communities, commercial housing, affordable housing and informal housing. These residential blocks are located in seven of the central, transitional and marginal districts of the city, which include Liwan, Yuexiu, Tianhe, Haizhu, Baiyun, Panyu and Huangpu Districts (Figure 2).

### 2.3. Data

#### 2.3.1. Data Collection and Participants’ Data

Data for this study were collected in August 2017 through a questionnaire survey called the “Survey of Residents’ Daily Activity and Community Integration in Guangzhou”. Specifically, the questionnaire survey ran from March 2017, and lasted about five months. During this period, some trained interviewers who were experienced employees of a professional survey research company in Guangzhou were hired. These interviewers received training through our detailed explanation of the questionnaire and the reasons for asking those questions in the context of the study, undertaking formal investigation and then collecting final questionnaire data in August 2017. The questionnaire survey was approved by Sun Yat-sen University (SYSU), and supported by grants from the National Natural Science Foundation of China (41522104), and all participants gave informed consent. Respondents in the survey were proportionally selected from the adult residents in the 11 selected residential blocks in Guangzhou based on the size of the permanent population of each block reported in the Sixth National Census of China. Each questionnaire was administered by a trained interviewer in a face-to-face interview with a participant, and it took about 30–40 min to fill out. A total of 1003 valid and usable questionnaires were finally obtained. 

Individual-level data items solicited through the questionnaires include personal characteristic (demographic and socioeconomic characteristics), residential and employment information, physical activity, self-reported health conditions, social interactions, and activity logs. 

(i) Demographic and socioeconomic characteristics—Demographic indicators solicited through the questionnaires include gender, age, and marital status. The proportions of males and females in the sample are quite balanced. The proportion of young people (between the age of 19 and 44) is noticeably higher (75.37%; note that juveniles under 19 and elderly people over 59 were excluded from the study). In addition, education level and personal monthly income, which are highly related to socioeconomic status, were also obtained from the participants.

(ii) Physical activity indicators—Physical activity level was assessed mainly by their duration, frequency, and intensity [33,37]. The duration and frequency of physical activity over the past week were self-reported by participants and include three types of physical activity (PA): Brisk walking (for recreational and transportation purposes), moderate PA (dancing, playing bowling/ping-pong/badminton, and so on) and vigorous PA (aerobic exercise, running, fast cycling, swimming, playing basketball/football, and so on). The intensity of weekly physical activity is measured by metabolic equivalents (METs). Metabolic equivalents are equal to total brisk walking minutes × 3.5 + total moderate PA minutes × 4.0 + total vigorous PA minutes × 8.0 (International Physical Activity Questionnaire, IPAQ) [37,56,57]. Weekly metabolic equivalents (METs) are used to assess whether the participants have met the physical activity recommendation (>600 MET-min/week). Low-level PA (0–600 MET-min/week) is defined as not meeting the recommendation, intermediate-level PA (600–1500 MET-min/week) and high-level PA (>1500 MET-min/week) are defined as meeting the recommendation and exceeding the recommendation respectively [58]. Specifically, this recommendation (>600 MET-min/week) could be regarded as a standard for an individual to engage in his or her physical activity. For example, low-level PA that doesn’t meet the recommendation chronically may lead to adverse individual health outcomes. Conversely, people’s morbidity and mortality will drop significantly as they increase their physical activity from a low level to an intermediate level or a high level.

(iii) Health indicators—The definition of health by the WHO in 1948 is “A state of complete physical, mental and social well-being and not merely the absence of disease or infirmity.” Thus, this study focuses on the three dimensions of physical health, mental health, and social health [59]. Physical health refers to the state that people has a strong and healthy physique, as well as a better self-protection ability to reduce harm and restore an (adapted) equilibrium [59]. Information about participants’ individual subjective feeling of physical health status was obtained in the survey using the *MOS 36-Item Short-Form Health Survey (SF-36*, *items 1*, *4*, *and 7)* [60], which has been widely used in previous studies. Mental health is defined as a state of emotional well-being, in which individual can recognize his or her own potential, cope with stressful situations effectively, work productively and fruitfully, and make a contribution to her or his community. The *World Health Organization’s Five Well-Being Indexes (WHO-5)* [61], which has short and positively worded items, is one of the most widely used instruments for assessing people’s subjective mental health. Social health refers to the ability of an individual to have a good interpersonal relationship and social adaptation. For this research, the five questions on social health used in the survey were derived from the scales used in previous studies (*Social Cohesion and Trust Scale* [62], *Social Wellbeing Scale* [63] and *Social Support List-Interactions (SSL-I)* [64]), and their reliability and validity have been confirmed to be excellent [39,65]. All of these self-evaluation indicators are described qualitatively through a 5-point Likert scale ranging from “poor” to “excellent.”

#### 2.3.2. Activity Space of Participants

**Figure 3:**
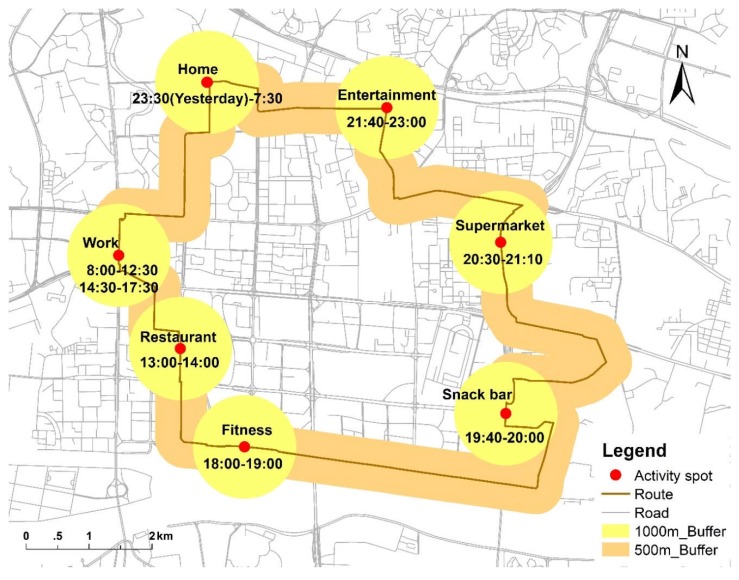
Construction of individual activity space using two types of buffer areas.

Activity space, which is the area containing all locations where an individual undertakes his or her daily activities [52,66], is used to delineate individual contextual units in this study. In the survey, 1003 participants were interviewed and a total of 14,439 items were recorded in their activity logs for a weekday, so there are approximately 14.4 activities recorded for each participant. These items include activity locations or stay points (residences, workplaces, restaurants, shopping places, fitness places, entertainment places and so on) and travel characteristics like origin, destination, transportation mode and time spent. Among these activity spaces, the top three where participants spent most time were residence (54.99%), workplace (32.88%) and travel (8.64%) (Table 2). Based on these detailed activity log data, the activity space for each participant was delineated using actual individual trajectory reported by participants. Note that Kwan et al. [52] have compared seven different methods for delineating people’s activity space and found that different methods may lead to different individual exposure level and health outcomes. Since different methods for delineating activity space have different strengths and weaknesses, we used a hybrid method in this study to integrate two elements of participants’ daily activities and mobility to assess their greenspace exposure: The activity space of each participant was constructed using two types of buffers based on their activity locations or stay points and travel behaviors, respectively. According to the buffer sizes used in previous studies [67,68,69], a 1000 m-buffer was used around each stay point and a 500 m-buffer was used for travel routes (Figure 3). Due to the different durations that each participant spent at different activity locations, the person’s exposure to greenspace would also change over time. Therefore, the effect of time on greenspace exposure also needs to be considered when constructing the activity space. In this research, individual daily greenspace exposure was more accurately assessed based on the proportion of time spent at different activity locations. The formulae and computing steps are given in Section 2.3.3 below. 

#### 2.3.3. Greenspace Data and Exposure Assessment

Greenspace data used in this study were automatically extracted and calculated from remote sensing images covering Guangzhou in November 2015 using ENVI 5.2 (Palm Bay, FL, USA) and ArcGIS 10.3 (Redlands, CA, USA). These remote sensing images, with a spatial resolution of 2 m, are obtained from the Gao Fen-1 (GF-1) satellite, the first satellite of China’s High-Resolution Earth Observation System (CHEOS). Using these remote sensing images, three objective indicators (vegetation coverage, physical activity site coverage, and accessibility to the nearest greenspace) used in previous studies [36,37] were selected for measuring participants’ exposure to greenspace in the study area. Among these indicators, vegetation coverage and physical activity site coverage were calculated based on the time-weighted average method [70,71]. The three objective indicators are described as follows.

(1) Vegetation coverage: This is the time-weighted proportion of the area of vegetation that is within the activity space buffers of a participant.

(1) $$Vegetation\;coverage=\left(\frac{S_{v1}}{S_{b1000}}\times \frac{t_{1}}{24}+\frac{S_{v2}}{S_{b1000}}\times \frac{t_{2}}{24}+\ldots +\frac{S_{vn}}{S_{b1000}}\times \frac{t_{n}}{24}\right)+\left(\frac{S_{vt}}{S_{b500}}\times \frac{t_{t}}{24}\right)\;,$$

(2) $$t_{1}+t_{2}+\ldots +t_{n}+t_{t}=24\left(h\right),$$

where $S_{b1000}$ is the area of 1000 m-buffer; $S_{v1}$ is the area of vegetation coverage in the first activity space buffer, $S_{vn}$ is the area of vegetation coverage in the *n*th activity space buffer, and so on; $t_{1}$ is the time the participant spent in the first activity space, $t_{n}$ is the time the participant spent in the *n*th activity space, and so on; $S_{b500}$ is the area of 500 m-buffer; $S_{vt}$ is the area of the vegetation coverage in the travel route buffer; $t_{t}$ is the time the participant spent in the travel route.

(2) Physical activity site coverage: This is the time-weighted proportion of the area of physical activity sites (parks, squares, outdoor playgrounds, and so on) that can be accessed within a participant’s activity space buffers.

(3) $$\begin{array}{l} \;Physical\;activity\;site\;coverage\\ \;=\left(\frac{S_{pas1}}{S_{b1000}}\times \frac{t_{1}}{24}+\frac{S_{pas2}}{S_{b1000}}\times \frac{t_{2}}{24}+\ldots +\frac{S_{pasn}}{S_{b1000}}\times \frac{t_{n}}{24}\right)+\left(\frac{S_{past}}{S_{b500}}\times \frac{t_{t}}{24}\right),\; \end{array}$$

where $S_{pas1}$ is the area of physical activity site in the first activity space buffer, $S_{pasn}$ is the area of physical activity site in the *n*th activity space buffer, and so on; $S_{past}$ is the area of physical activity site in the travel route buffer.

(3) Accessibility to the nearest greenspace: This is the average of the sum of the distances between each activity site of a participant to the nearest greenspace in the respective activity space buffers.

(4) $$\;Accessibility\;to\;the\;nearest\;greenspace=\left(\frac{D_{1}+D_{2}+\ldots +D_{n}}{n}\right),\;$$

where $D_{1}$ is the distance from the first activity site to its nearest greenspace in the first activity space buffer, $D_{n}$ is the distance from the *n*th activity site to its nearest greenspace in the *n*th activity space buffer, and so on; *n* is the number of activity space buffer. These three greenspace exposure indicators were used in this study to capture various forms of greenspace exposure for the participants. 

### 2.4. Structural Equation Modeling

As a powerful method for examining the causal relationships among a set of variables, structural equation modeling has been widely used in the literature of health geography [72,73]. It can be used to estimate abstract concepts (such as health status) using measured variables, examine the complex causal relationships among variables using feedback loops, and improve the accuracy and credibility of model results by considering the influence of measurement error. Structural equation modeling is suitable for identifying the mediating effects of variables and thus was used in this study. 

Before constructing the structural equation model (SEM), the reliability and validity of the variables were verified by using Cronbach’s Alpha and factor analysis in order to make the model results more convincing. The results suggest that variables selected in this study have a relatively high reliability (Cronbach’s Alpha is 0.726 (≥0.700)) and validity (Kaiser-Meyer-Olkin (KMO) is 0.779 (>0.700) and Sig. is 0.000 (<0.05)). 

Given that this study focuses mainly on the direct effects of greenspace exposure on health and the indirect effects of greenspace exposure on health through physical activity, personal characteristics are taken mainly as control variables in the SEM (Table 3). Greenspace exposure, physical activity, and health status are the exogenous variable, mediator variable, and endogenous variable respectively in the model (Table 4).

## 3. Results

### 3.1. Model Testing

**Figure 4:**
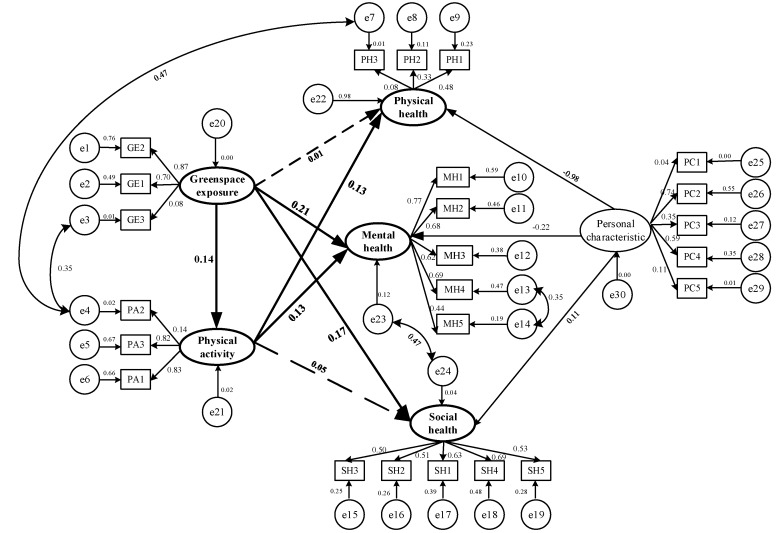
Effects of daily greenspace exposure on health.

The SEM is constructed and revised using AMOS 21.0. It is found that the SEM presented in Figure 4 is an ideal research model with high goodness-of-fit and stability through the analysis of the structural equation model’s matching degree (Table 5). Besides, the measuring results of the SEM’s paths suggest that five paths in the hypotheses are verified (C.R. > 1.96, *p* < 0.05). As shown in Table 6, these verified paths indicate that greenspace exposure has a significant positive effect on mental health, social health, and physical activity, thus retaining H2, H3, and H4, respectively. Meanwhile, the paths from physical activity to physical health and mental health are significant, respectively, retaining the hypotheses (H5 and H6) that physical activity has a positive effect on physical health and mental health. Although greenspace exposure has an effect on physical health, and physical activity has a positive influence on social health, the effects of these two paths are not statistically significant (*p* > 0.05; H1 and H7 are invalid). Thus, the effect of greenspace exposure on physical health and the effect of physical activity on social health are both considered 0.00 in further analysis. The specific effect relationships between the latent variables are shown in Table 7.

### 3.2. Effects of Daily Greenspace Exposure on Health

#### 3.2.1. Direct Effects

The correlation coefficients between greenspace exposure and its measured variables (vegetation coverage [GE1], physical activity site coverage [GE2] and accessibility to the nearest greenspace [GE3]) are 0.70, 0.87 and 0.08 respectively (Figure 4), suggesting that “vegetation coverage (GE1)” and “physical activity site coverage (GE2)” are more closely related to participant’s daily greenspace exposures. As shown in Table 7, greenspace exposure has a direct positive effect on mental health (0.21) and social health (0.17). However, greenspace exposure is not associated with physical health (0.00). These direct effects on the three dimensions of health indicate that participants’ psychological condition and social interactions are significantly better for participants who were exposed to more greenspace than those who were exposed to more greenspace-poor areas. In addition, the impact of greenspace exposure on mental health is more obvious than its impact on social health. 

#### 3.2.2. Indirect Effects

The indirect effects of daily greenspace exposure on individual health are realized through physical activity, the mediator variable. The correlation coefficients between physical activity and its measured variables (duration [PA1], frequency [PA2] and intensity [PA3]) are 0.83, 0.14, and 0.82, respectively (Figure 4), suggesting that “duration (PA1)” and “intensity (PA3)” of physical activity have great influences on the level of physical activity undertaken by participants. Daily greenspace exposure level has a strong relationship with physical activity level (*p* < 0.01) (Table 6). It is similar to what was found in previous studies that greenspace provides the beautiful environment and comfortable space for physical activity and could effectively alleviate the decline in physical activity level, due to a lack of venues [74,75]. Besides, the correlation between “accessibility to the nearest greenspace (e3)” and “frequency (e4)” shows that an increase in the accessibility of greenspace may enhance the frequency of people’s physical activity (Figure 4). Physical activity has a significant positive effect on physical health (0.13) and mental health (0.13), as shown in Table 6. In particular, the correlation between “frequency (e4)” and “self-rated physical health (e7)” indicates that the higher the frequency of a participant’s physical activity, the better is her or his physical health (Figure 4). However, physical activity is not associated with social health (0.00), so this outcome is excluded from further analysis. 

As shown in Table 7, the indirect effects of daily greenspace exposure on participants’ physical health and mental health through physical activity are 0.018 and 0.018, respectively. The results indicate that the indirect effect of daily greenspace exposure on mental health (0.018) is equal to that on physical health (0.018), but is greater than that on social health (0.00). In contrast, the direct effect of daily greenspace exposure on social health (0.17) is less than that on mental health (0.21), but is greater than that on physical health (0.00). By comparing the results of indirect effects and direct effects of daily greenspace exposure on health, it can be observed that the effects of daily greenspace exposure on physical health and social health changed greatly after adding the mediating variable of physical activity, indicating that physical activity plays a mediating role in the relationship between greenspace exposure and health. 

#### 3.2.3. Total Effects

The total effects (direct effects + indirect effects) of daily greenspace exposure on physical health, mental health, and social health are 0.018, 0.228, and 0.17, respectively (Table 7), all of which have increased when compared with the direct effects. 

Among the three dimensions of health, daily greenspace exposure level has the most obvious effect on mental health. This indicates that daily greenspace exposure plays a primary role in improving participants’ mental health, likely through an increase in the quantity and quality of greenspace in their activity space. Specifically, people could release stress and tension, mitigate negative emotions and create a relaxed and pleasant mental state through frequent contact with greenspace. 

Although daily greenspace exposure has the lowest indirect effect on social health, its total effect on social health is second, due to its higher direct effect. This suggests that natural environment provides more opportunities for people to engage in physical activity with families and neighbors, which promotes interpersonal relationships, fosters social interactions, strengthens community cohesion, and enhances individual well-being. 

Notably, daily greenspace exposure has the lowest total effect on physical health, due to the fact that its direct effect on physical health is the lowest. The main reason resulting in its lowest direct effect on physical health is that this study only considers the coverage of greenspace and ignores other factors, such as the diversity of plant species, landscaping design and layout, and so on. In contrast, the indirect effect of daily greenspace exposure on physical health is higher, indicating that promoting physical health through physical activity may yield better outcomes than relying on the direct effects. For instance, greenspace with high accessibility and attractive surroundings can help stimulate people’s interest in physical activity, which in turn reduces the incidence of diseases and helps maintain health through higher levels of physical activity. 

## 4. Discussion

Urban greenspace planning is a crucial issue in the context of rapid urbanization and sustainable development, as greenspace helps to support physical activity and improve individual health outcomes. Recent research has underlined the importance of planning and management of greenspace, especially in megacities, due to the huge populations and scarcity of space [76,77,78].

### 4.1. Urban Greenspace Planning Implications

Urban greenspace exposure makes a great contribution to counteracting people’s sedentary lifestyle, increasing their physical activity and improving their health status. However, there is a general underestimation of the value of daily greenspace exposure in urban planning and park management in China. Therefore, conducting research in this area and applying the findings to improve the planning and design of greenspace in urban areas has great significance to urban sustainability and healthy living. In order to further promote the beneficial influence of greenspace on health outcomes, residents should be advised to heighten their environmental protection consciousness and increase their utilization rate of greenspace. In addition, urban planners should take more measures in constructing greenspace and building ecological cities so as to increase people’s greenspace exposure. These specific measures include increasing the proportion of greenspace in urban areas, and promoting supporting facilities and services like seats and outdoor exercise or fitness equipment. Finally, relevant government departments should establish and improve laws and regulations to protect public health through constructing a green environment for promoting health and formulating and implementing health education action plan. 

### 4.2. Limitations

One limitation of this research is that greenspace exposure indicators included only objectively measured variables, which precluded the ability to draw conclusions about the influence of subjective assessment of greenspace exposure on health. Subjective assessment should be coupled with objectively measured data in future studies, such as the evaluation of the quantity and quality of one’s activity space, the utilization rate of greenspace and subjective assessment of sanitary condition. 

Another limitation of this study relates to the survey of mental health, which only reflects the overall state of mind in the recent period. However, mental state (pleasant, stressful, anxious, and so on) and emotions (positive feelings, negative feelings, and so on) will also change momentarily depending on different events that people experience in their daily life. Future research may shed new light on the moderating effects of greenspace exposure on changes in people’s mental states that are affected by the stressful events or daily emergencies they experience. For instance, respondents may be asked to wear global positioning systems (GPS) to track their movement patterns in a more objective manner and list all activities they are engaged in and how they felt during each activity through responding to real-time prompts based on ecological momentary assessment (EMA) methods. In future work, conducting studies with these additional components has potential to help improve research results. 

## 5. Conclusions

The objective of this study is to examine the relationship between daily greenspace exposure and individual health from the point of view of human mobility and the uncertain geographic context problem. The results indicate that daily greenspace exposure directly influences participants’ health and indirectly affects their health status through the mediating effect of physical activity. Specifically, the direct effect of daily greenspace exposure on mental health is more significant than its direct effect on social health, while such direct effect on physical health is not obvious. The indirect effect of daily greenspace exposure on mental health is similar to that on physical health, but such indirect effect on social health is not remarkable. In addition, the total effect of daily greenspace exposure on mental health is more obvious than its total effect on social health. However, the total effect of daily greenspace exposure on physical health is not significant. On the whole, a higher level of individual daily greenspace exposure in the study area is related to better physical activity and overall health. Daily greenspace exposure primarily helps to improve participants’ mental health and relieve their negative feelings, and then promote their social health and strengthen social cohesion, and enhance their physical health and reduce the incidence of diseases to a lesser extent.

## Figures and Tables

**Table 1:** Hypotheses for this study.

Hypotheses
H1 Daily greenspace exposure has a significant positive effect on physical health.
H2 Daily greenspace exposure has a significant positive effect on mental health.
H3 Daily greenspace exposure has a significant positive effect on social health.
H4 Daily greenspace exposure has a significant positive effect on physical activity, which plays a mediating role in the relationship between daily greenspace exposure and individual health.
H5 Physical activity has a significant positive effect on physical health.
H6 Physical activity has a significant positive effect on mental health.
H7 Physical activity has a significant positive effect on social health.

**Table 2:** Time spent of the study participants on daily activity (N = 1003).

Daily Activity	Time Spent (h)	Per Capita Time Spent (h)	Percentage in a Weekday
Residence	13,236.08	13.20	54.99%
Work	7914.25	7.89	32.88%
Dining (in restaurant)	364.17	0.36	1.51%
Shopping	106.87	0.11	0.44%
Fitness (in fitness place)	86.93	0.09	0.36%
Entertainment	98.53	0.10	0.41%
Travel	2079.67	2.07	8.64%
Other	183.67	0.18	0.76%
Total	24,070.17	24.00	100.00%

**Table 3:** Demographic and socioeconomic characteristics of the study participants (N = 1003).

Personal Characteristic	Code	Variable	Percent (%)
Gender	PC1	Male	49.95
		Female	50.05
Age (years)	PC2	Young people (19–44)	75.37
		Middle-aged people (45–59)	24.63
Marital status	PC3	Married	80.06
		Single	19.94
Education	PC4	Primary school or lower	0.10
		Junior high school degree	6.28
		Senior high school degree	27.52
		Bachelor degree	65.20
		Master degree or higher	0.90
Personal monthly income (RMB)	PC5	≤2999 Yuan	1.20
		3000–4999 Yuan	32.10
		5000–8999 Yuan	48.55
		9000–11,999 Yuan	7.48
		≥12,000 Yuan	10.67

**Table 4:** Variables of the structural equation model.

Type	Latent Variable	Measured Variable	Code
Exogenous variable	Greenspace exposure	Vegetation coverage	GE1
		Physical activity site coverage	GE2
		Accessibility to the nearest greenspace	GE3
Mediator variable	Physical activity	Duration	PA1
		Frequency	PA2
		Intensity	PA3
Endogenous variable	Physical health	How much bodily pain have you had during the past four weeks?	PH1
		During the past four weeks, have you had any problems with your work or other regular daily activities as a result of your physical health?	PH2
		In general, what would you say your physical health is?	PH3
	Mental health	I have felt cheerful and in good spirits	MH1
		I have felt calm and relaxed	MH2
		I have felt active and vigorous	MH3
		I woke up feeling fresh and rested	MH4
		My daily life has been filled with things that interested me	MH5
	Social health	People around here are willing to help their neighbors	SH1
		This is a close-knit neighborhood	SH2
		People in this neighborhood can be trusted	SH3
		People in this neighborhood get along well with each other	SH4
		People in this neighborhood can handle questions together	SH5

**Table 5:** Analysis of the structural equation model’s matching degree.

	CMIN/DF	GFI	RMR	RMSEA	AGFI	PNFI	PCFI
Suggested values	≤5	>0.90	<0.05	<0.08	>0.90	>0.50	>0.50
Correction model	4.790	0.913	0.035	0.061	0.892	0.694	0.723

**Table 6:** Test results of the causal paths of the SEM.

Relationship between Variables	Path Coefficient ^a^	C.R.	*p*	Consequence
Greenspace exposure → Physical health	-	-	-	H1 Invalid
Greenspace exposure → Mental health	0.21	5.344	***	H2 Valid
Greenspace exposure → Social health	0.17	3.968	***	H3 Valid
Greenspace exposure → Physical activity	0.14	3.213	**	H4 Valid
Physical activity → Physical health	0.13	2.156	*	H5 Valid
Physical activity → Mental health	0.13	3.457	***	H6 Valid
Physical activity → Social health	-	-	-	H7 Invalid

*** *p* < 0.001, ** *p* < 0.01, * *p* < 0.05; ^a^ Standardized path coefficients.

**Table 7:** Effect relationships between the latent variables of the SEM.

Total Effect	Direct Effect	Indirect Effect
Greenspace exposure → Physical health	Greenspace exposure → Physical health	Greenspace exposure → Physical activity → Physical health
(0.018)	(0.00)	(0.018)
Greenspace exposure → Mental health	Greenspace exposure → Mental health	Greenspace exposure → Physical activity → Mental health
(0.228)	(0.21)	(0.018)
Greenspace exposure → Social health	Greenspace exposure → Social health	Greenspace exposure → Physical activity → Social health
(0.17)	(0.17)	(0.00)

Greenspace exposure → Physical activity → Physical health: It is a path that greenspace exposure affects physical health indirectly by affecting physical activity. In this indirect path, there are two direct paths: “Greenspace exposure → Physical activity” (0.14) and “Physical activity → Physical health” (0.13), which means that physical activity as a mediator connects the other two variables (greenspace exposure and physical health) and drives this indirect path. Thus, the indirect effect of greenspace exposure on physical health is 0.14 × 0.13 ≈ 0.018 (three decimal places).
